# Gene Flow and Hybridization between Numerically Imbalanced Populations of Two Duck Species on the Subantarctic Island of South Georgia

**DOI:** 10.1371/journal.pone.0082664

**Published:** 2013-12-18

**Authors:** Kevin G. McCracken, Robert E. Wilson, Anthony R. Martin

**Affiliations:** 1 Institute of Arctic Biology, University of Alaska Museum, and Department of Biology and Wildlife, University of Alaska Fairbanks, Fairbanks, Alaska, United States of America; 2 Centre for Remote Environments, University of Dundee, Dundee, United Kingdom; Aarhus University, Denmark

## Abstract

Hybridization is common between species of animals, particularly in waterfowl (Anatidae). One factor shown to promote hybridization is restricted mate choice, which can occur when 2 species occur in sympatry but one is rare. According to the Hubbs principle, or "desperation hypothesis," the rarer species is more likely to mate with heterospecifics. We report the second of 2 independent examples of hybridization between 2 species of ducks inhabiting island ecosystems in the Subantarctic and South Atlantic Ocean. Yellow-billed pintails (*Anas georgica*) and speckled teal (*Anas flavirostris*) are abundant in continental South America, where they are sympatric and coexist in mixed flocks. But on South Georgia, an isolated island in the Subantarctic, the pintail population of approximately 6000 pairs outnumbers a small breeding population of speckled teal 300∶1. Using 6 genetic loci (mtDNA and 5 nuclear introns) and Bayesian assignment tests coupled with coalescent analyses, we identified hybrid-origin speckled teal alleles in 2 pintails on South Georgia. While it is unclear whether introgression has also occurred into the speckled teal population, our data suggest that this hybridization was not a recent event, but occurred some time ago. We also failed to identify unequivocal evidence of introgression in a much larger sample of pintails and speckled teal from Argentina using a 3-population "Isolation-with-Migration" coalescent analysis. Combined with parallel findings of hybridization between these same 2 duck species in the Falkland Islands, where population ratios are reversed and pintails are outnumbered by speckled teal 1:10, our results provide further support for the desperation hypothesis, which predicts that scarcity in one population and abundance of another will often lead to hybridization. While the South Georgia pintail population appears to be thriving, it's possible that low density of conspecific mates and inverse density dependence (Allee effect) may be one factor limiting the reproductive output of the speckled teal population, and this situation may persist unless speckled teal increase in abundance on South Georgia.

## Introduction

Interspecific hybridization is an important mechanism of lineage diversification and adaptation in plants [1], [2], [3], and it has also been shown to be an important evolutionary force in animals [4], [5], [6]. Birds are no exception; at least one in ten species is known to hybridize [7], [8], [9], [10]. The waterfowl (Anatidae) comprise more than half of known avian hybrids [11], [12], [13]. Numerous factors have been implicated in the ability of the Anatidae to hybridize [12], [14], including Haldane's [15] rule. One factor in particular is that hybridization is encouraged by restricted mate choice, and is therefore common in areas where two species occur in sympatry but one species is rare [14]. This concept was first formalized by Hubbs [16]: "Great scarcity of one species coupled with the abundance of another often leads to hybridization: the individuals of the sparse species seem to have difficulty in finding their proper mates." Hubbs referred to this principle as the "desperation hypothesis," for which empirical support has now been found among numerous species of birds, including waterfowl [14], [17].

Here, using multilocus genetic data, we report the second of two examples of interspecific hybridization between two common species of waterfowl that exist in widespread sympatry throughout southern South America, but which have hybridized in numerically imbalanced situations, where they inhabit island ecosystems in the Subantarctic and South Atlantic Ocean. In the first case, previously reported by McCracken & Wilson [17], a common species, the speckled teal (*Anas flavirostris*), was found to have hybridized with a much less abundant population of yellow-billed pintails (*Anas georgica*) in the Falkland Islands, offering an empirical example of support for Hubbs’ [16] principle, the “desperation hypothesis”.

Using data from six genetic loci, Bayesian assignment tests, and coalescent models, we reveal here that these same two species have hybridized on the subantarctic island of South Georgia, where the ratios are reversed and the pintail population of 6,000 pairs outnumbers a small breeding population of speckled teal by approximately 300:1. These data thus illustrate yet another example whereby alleles from an uncommon species have introgressed into the population of the significantly more abundant species. We further speculate about factors leading to the development of the current and future avifauna of South Georgia, as glaciers continue to recede from this heavily glaciated island and expose new habitat suitable for additional species of waterfowl. We also consider how a low density of conspecific mates, which has likely promoted interspecific hybridization, might contribute to a type of inverse density dependence known as the Allee [18] effect that can limit the growth rate of very small populations.

## Materials and Methods

### Study Site and Taxa

The island of South Georgia (54.0–55.0°S, 35.5–38.5°W) lies isolated in the South Atlantic Ocean 1,300 km east-southeast of the Falkland Islands. The closest continental land areas are South America and the Antarctic Peninsula, at distances of 1,800 and 1,500 km, respectively. The island sits at the junction of the Pacific and Atlantic plates on the Scotia Arc, a mid-oceanic ridge that extends east from Tierra del Fuego. The island is very mountainous, with peaks rising to 2,935 m. Approximately 58% of the island is still covered with ice [19], [20], and glaciers have carved fjords and valleys on all sides of the island. Ice-free areas at lower elevations are dominated by tundra-like vegetation, peat bogs, and small ponds, with a notable absence of trees and a low number of vascular plants [21]. The climate of South Georgia is cool and wet, with a mean annual temperature of 2.0°C, and its weather is dominated by polar cyclones that traverse the Southern Ocean [20].

Because it lies south of the Polar Frontal Zone in the Antarctic Circumpolar Current, South Georgia has long been of interest to glacial geologists and paleoclimate scientists [22]. Studies indicate that the island experienced extensive glaciation and that ice caps extended to the continental shelf [19], [23], [24], [25], [26]. While the maximum extent of the ice cover is not questioned, the timing of recent deglaciation prior to and through the Last Glacial Maximum (LGM) is still a matter of debate, as a variety of studies throughout the Antarctic, including South Georgia, have indicated that ice-free refugia likely harbored many plant and animal species through numerous glacial and interglacial cycles [27], [28], [29], [30], [31], [32].

Among these glacial survivors on South Georgia is an endemic, non-migratory subspecies of dabbling duck (Aves: Anatidae) called the South Georgia pintail (*Anas g. georgica*), which likely colonized the archipelago prior to the Last Glacial Maximum [32]. The South Georgia pintail is morphologically distinct in both body size (smaller) and plumage color (darker) from its closest relative, the yellow-billed pintail (*Anas g. spinicauda*), which ranges widely over southern South America and is the most common waterfowl species in that region, likely numbering more than one million individuals [33]. On South Georgia, by contrast, pintails number approximately 6,000 pairs [34], [35], [36], [37]. Like other endemic high-latitude island ducks, South Georgia pintails are predominately intertidal feeders, as freshwater ponds freeze and snow blankets the island to the shoreline during most of the year. Even during summer, individuals infrequently stray far from the coast, and because less than 50% of the island is deglaciated, available inland freshwater habitat is still limited. Although the Chiloe wigeon *(Anas sibilatrix*) has also been recorded on South Georgia, the only other duck species that has been reported breeding is the speckled teal (*Anas flavirostris*) [38], [39]. Whereas there are about 6,000 pairs of pintails, there are fewer than 20 pairs of speckled teal, causing an imbalanced ratio of approximately 300:1 between populations of these two species, which are relatively closely related congeners but not sister taxa [40].

### Specimen Collection, PCR, and DNA Sequencing

**Figure 1:**
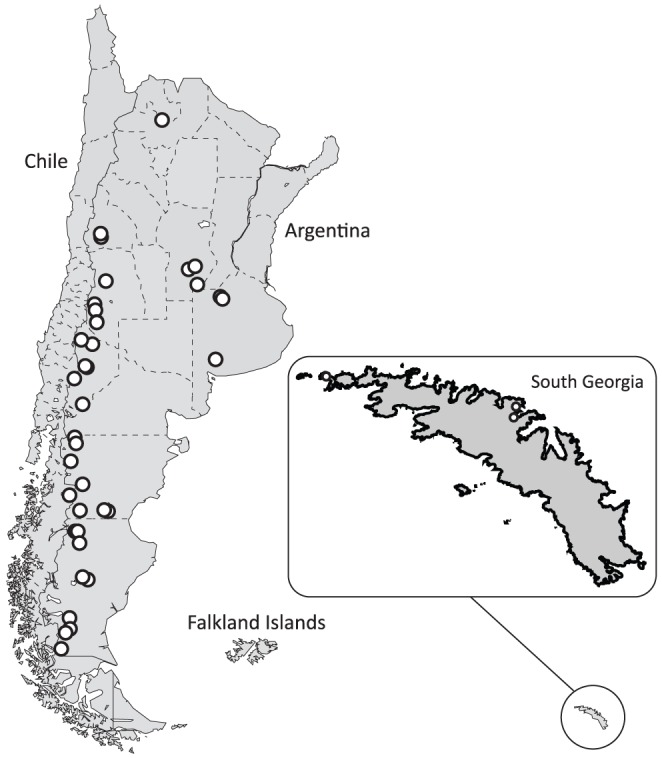
Map illustrating the localities of pintail samples on South Georgia and in Argentina (speckled teal localities are shown in figure 1 of McCracken & Wilson [17]).

South Georgia pintails were banded by ARM at various sites on the island between 1998 and 2002 (*n*  =  60; Fig. 1). Yellow-billed pintails (*n*  =  64; Fig. 1) and speckled teal (*n*  =  56) were collected by KGM and REW in Argentina between 2001 and 2005 (Fig. 1; see figure 1 in McCracken & Wilson [17]). No speckled teal were sampled on South Georgia due to their low abundance; speckled teal from Argentina were thus pooled with pintails from South Georgia and Argentina and used as a proxy for nonexistent samples of speckled teal from South Georgia. Vertebrate collecting activities were approved by the University of Alaska Fairbanks Institutional Animal Care and Use Committee (IACUC 02-01, 05-05) and by federal and provincial governments in Argentina and South Georgia (D.F.S. No. 3209/01, 13168/03, 13169/03, 20419/05, 20420/05). Total genomic DNA was isolated from web punches and muscle tissue, respectively, using standard protocols with DNeasy Tissue Kits (QIAGEN, Valencia, California). Six gene regions including the mitochondrial DNA (mtDNA) control region and five nuclear loci were sequenced using PCR and DNA sequencing protocols described in previously published manuscripts utilizing these sequences (Table 1; [17], [32], [41], [42]). Sequences and specimen voucher information, including geo-referenced localities, are available in GenBank (accession numbers [32] KC987596–KC987946, [41] FJ617817–FJ618512, [42] GQ269874-GQ269883, GQ269899-GQ269943, GQ270014- GQ270023, GQ270039-GQ270084, GQ270155-GQ270164, GQ270180-GQ270225, GQ270296-GQ270306, GQ270322-GQ270372, GQ270476-GQ270485, GQ270501-GQ270546, and [17] JN223305-JN223314, JN223330-JN223375).

**Table 1:** Genes sequenced and their chromosomal positions in the chicken genome.

Locus	Base pairs sequenced	Chicken chromosome
mtDNA control region (mtDNA)	977–981	mtDNA
Ornithine decarboxylase intron 5 (ODC1)	352	3
α enolase intron 8 (ENO1)	314	21
β fibrinogen intron 7 (FGB)	246	4
N-methyl D aspartate 1 glutamate receptor intron 11 (GRIN1)	328–330	17
Phosphoenolpyruvate carboxykinase intron 9 (PCK1)	345–351	20

Location in the chicken genome as defined by Hillier et al. [81].

### Allelic Phase Determination

The allelic phase of each nuclear sequence that was heterozygous at two or more nucleotide positions was determined using allele-specific priming and the software PHASE 2.1 [43]. PHASE uses a Bayesian algorithm to infer haplotypes from diploid genotypic data while incorporating recombination and the decay of linkage disequilibrium (LD) with genetic distance, and it is as effective or better than cloning for resolving highly polymorphic sequences [44], [45]. We first analyzed each composite sequence, the consensus of both alleles, using the default software settings (100 main iterations, 1 thinning interval, 100 burn-in) followed by 1,000 main iterations and 1,000 burn-in (-X10 option) for the final iteration. The PHASE algorithm was run five times (-x5 option) from different starting points, selecting the result with the best overall goodness of fit. For individuals with allele pair probabilities <80%, we then designed allele-specific primers to selectively amplify a single allele [46], [47]. The resulting haploid allele sequence was then subtracted from the diploid composite sequence to obtain the gametic phase of the second allele. Each data set was then analyzed five more times using PHASE and the additional known allele sequences (-k option). The gametic phase of each autosomal sequence was identified experimentally or with greater than 95% posterior probability for >96% of the individuals included in each data set.

### Identification of Introgressed Speckled Teal Alleles

Introgressed alleles were identified in the South Georgia pintail population by pooling the sequences from both species and comparing pintails to speckled teal as outlined in McCracken & Wilson [17]. Briefly, the same set of mtDNA and five nuclear loci were sequenced for both pintails and speckled teal, and all six loci yielded significant frequency differences between yellow-billed pintails and speckled teal (see table 3 in McCracken & Wilson [17]; Φ*_ST_* ranged from 0.11 to 0.94 between species). Two methods were then used to survey the South Georgia pintail population for heterospecific-origin alleles and to quantitatively test the hypothesis that speckled teal alleles have introgressed into the South Georgia pintail population.

First we used STRUCTURE 2.2 [48] to identify individual South Georgia pintails that might possess alleles introgressed from speckled teal. In the first step of the STRUCTURE analysis, a simple two-population model (*K*  =  2) was used for 60 South Georgia pintails and 56 speckled teal (excluding individuals from the Falkland Islands) using the admixture model (α  =  1) with independent allele frequencies (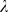  =  1) and no *a priori* population information (POPFLAG  =  0). In the second step, individuals with assignment probabilities >0.99 as determined in the first analysis were pre-assigned to their respective clusters corresponding to populations 1 and 2 (POPFLAG  =  1). The ancestry of individuals with assignment probabilities <0.99 in the first analysis was then reestimated (POPFLAG  =  0) using allele frequencies defined by individuals previously determined to have posterior probabilities >0.99. Information about the allele frequencies from pre-defined individuals was thus used to improve accuracy of inference about the admixture of unknown individuals. Using this information as a guide to identify putatively introgressed alleles, we then illustrated allelic networks for both species combined using the median-joining algorithm in the software NETWORK 4.6 ([49]; Fluxus Technology, Ltd.).

Second, we performed a three-population Isolation-with-Migration analysis in IMa2 [50], which allows for analysis of divergence and gene flow between two or more populations. IMa2 is useful in this regard because it offers the ability to quantitatively distinguish between patterns of allele sharing that have resulted from gene flow (or hybridization) versus those that have resulted from shared ancestral polymorphisms predating the split between two species or populations. In this case, we estimated the effective population size parameters (θ), gene flow rates (*M*), and times since divergence (*t*) between two populations of pintails and one population of speckled teal (Table 2). Because the IMa2 model assumes that all sequences are free from intralocus recombination, we tested for recombination using the four-gamete test [51] implemented in DNAsp 4.10 [52] and truncated each sequence to include the longest fragment with no apparent recombination (*R_M_*  =  0 in the four-gamete test). For ODC1 this included positions 1–151, ENO1 positions 1–172, GRIN 1 positions 75–178, and PCK1 positions 1–254. Five nuclear loci (no mtDNA) were included in this analysis, and the HKY substitution model was used in the IMa2 analysis, as opposed to the infinite-sites model because all six loci in the two-species data set possessed three or more alleles at one or more sites. IMa2 was first run with wide uninformative priors. Analyses were then conducted with more narrow uniform priors that encompassed the full posterior distributions from the preliminary runs (θ  =  5, *M*  =  100, and *t*  =  2. The Markov chain Monte Carlo was run five times independently for 12 million steps, sampling the posterior distribution every 150 steps, with a burn-in of 500,000 steps. All runs included 20 chains with a geometric heating scheme.

**Table 2:** Estimated parameters in the three-population IMa2 analysis.

Parameter	Symbol	Population/divergence/migration
Population size parameter (4*N_e_*μ)	θ*_SP_*	Argentine pintails
	θ*_GE_*	South Georgia pintails
	θ*_FL_*	Speckled teal
	θ*_0_*	𝚯 ancestral at *t_0_*
	θ*_1_*	θ ancestral at *t_1_*
Time since divergence (*t*)	*t_0_*	Between South Georgia pintails and Argentine pintails
	*t_1_*	Between pintails and speckled teal
Migration (*m/*μ)	*M_SP-GE_*	Into Argentine pintails from South Georgia pintails
	*M_GE-SP_*	Into South Georgia pintails from Argentine pintails
	*M_SP-FL_*	Into Argentine pintails from speckled teal
	*M_FL-SP_*	Into speckled teal from Argentine pintails
	*M_GE-FL_*	Into South Georgia pintails from speckled teal
	*M_FL-GE_*	Into speckled teal from South Georgia pintails

To quantitatively test whether speckled teal alleles had introgressed into pintails or vice versa, we examined the resulting posterior distributions for the four scaled gene flow rate parameters. Estimates of *M_SP-FL_*, *M_FL-SP_*, *M_GE-FL_*, or *M_FL-GE_* (see definitions in Table 2) with a lower 95% highest posterior density (HPD) that did not overlap zero (non-zero interspecific gene flow) were interpreted as quantitatively strong evidence for hybridization. Alternatively, estimates of *M* that overlapped zero could not be interpreted as strong evidence of interspecific gene flow, but would be more likely to result from shared ancestral polymorphisms predating the split between the pintail and speckled teal lineages.

## Results

### Speckled Teal Alleles Introgressed into the South Georgia Pintail Population

**Figure 2:**
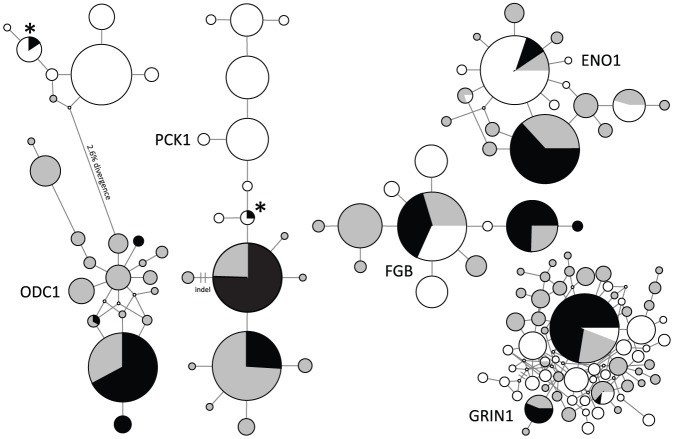
Networks for five nuclear loci sequenced from pintails and speckled teal. Alleles sampled from South Georgia pintails are illustrated in black, and alleles sampled from yellow-billed pintails in Argentina are illustrated in gray. Alleles samples from speckled teal are shown in white. Circle area is proportional to the total number of alleles.

Two South Georgia pintails that were heterozygous at the ODC1 locus possessed one allele each that were identical and 2.6% divergent from other pintails alleles (Fig. 2). Upon comparison to 56 speckled teal, these alleles were identical to the third most common speckled teal allele, which occurred in 11 individuals from Argentina (Fig. 2). A third pintail from South Georgia that was heterozygous at the PCK1 locus shared one allele with three specked teal. Finally, one pintail from Argentina (KGM 750) possessed a private singleton ODC1 allele that clustered with the speckled teal haplotype group (1 base pair divergent from the most similar speckled teal allele, but 2.6% divergent from the pintail cluster; see Fig. 2). No evidence of interspecific allele sharing was observed at other loci, including mtDNA. All South Georgia pintails possessed pintail mtDNA (see figure 2 in McCracken et al. [32]).

**Figure 3:**
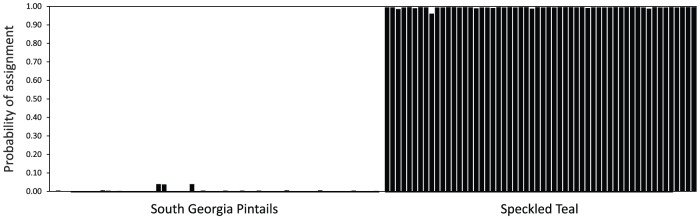
Assignment probabilities for South Georgia pintails and speckled teal for *K*  =  2 populations.

The STRUCTURE analysis corroborated these results. The three pintails from South Georgia described above had assignment probabilities of 0.959–0.961 (Fig. 3), whereas all other pintails on South Georgia were assigned to the island population with *P* > 0.99. Three South Georgia pintails thus exhibited evidence consistent with a single introgressed speckled teal allele.

**Figure 4:**
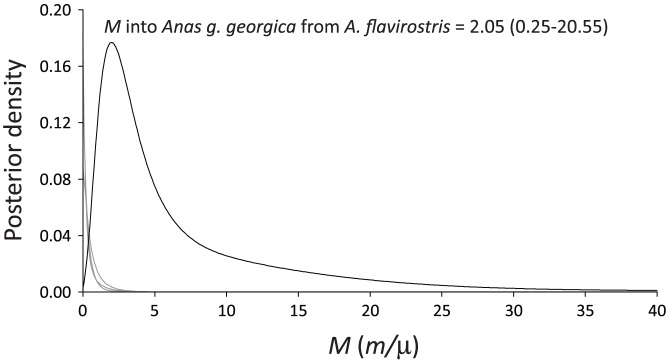
Gene flow estimates and the 95% highest posterior density (HPD) for nuclear DNA from the three-population IMa2 analysis between pintails and speckled teal. Gene flow from speckled teal into the South Georgia pintail population was positive. All other between-species gene flow parameter estimates peaked at zero.

The three-population IMa2 analysis supports the conclusion that speckled teal alleles have introgressed into the South Georgia pintail population. Gene flow from speckled teal into the South Georgia pintail population was greater than zero (*M_GE-FL_*  =  2.05, 95% HPD  =  0.25–20.55; Fig. 4), whereas the gene flow estimate in the opposite direction (South Georgia pintails → speckled teal) peaked at zero (*M_GE-FL_*  =  0.00, 95% HPD  =  0.00–1.35). Gene flow between pintails and speckled teal in Argentina also could not be distinguished from zero (Fig. 4). Considering the small population size of South Georgia pintails (θ*_GE_*  =  0.0125, 95% HPD  =  0.0075–0.1475), the long term, average effective number of speckled teal immigrating into the South Georgia pintail population is probably less than one per generation (4*N_e_m*  =  0.03).

## Discussion

### Distinguishing Hybrid Alleles from Shared Ancestral Polymorphisms

Species-level polyphyly can arise for multiple reasons, so the probability that a given allele has introgressed must be evaluated carefully against the possibility that the allele is a shared polymorphism that predates the split of two species [53], [54], [55], [56]. Furthermore, it is common for shared alleles to be retained across species boundaries, especially for populations that diverged recently and for nuclear DNA, which has a four-fold greater effective population size than mitochondrial DNA and is slower to sort to reciprocal monophyly [57], [58], [59].

South Georgia pintails and speckled teal are not sister taxa [40], and as our previous study indicated, the stem lineages giving rise to their respective clades likely diverged several million years ago [17]. Numerous alleles were nonetheless shared between species at all five nuclear loci, but not for mtDNA (Fig. 2). For three nuclear loci (ENO1, FGB, and GRIN1), the alleles that were shared between species were among the most common alleles, and net divergence was minimal. Shared alleles also occupied central positions in the haplotype networks, suggesting that they are ancestral. While drift and founder events can cause low frequency, non-ancestral alleles to become common, particularly in the island population during the process of colonization, this is unlikely to be the case in the continental populations, which have large effective population sizes.

In contrast to these other loci, ODC1 yielded two distinct clusters of alleles that were 2.6% divergent, and two pintails from South Georgia possessed one allele each that were identical to alleles found in speckled teal but not found in pintails in Argentina. One South Georgia pintail also possessed a PCK1 allele that was identical to a speckled teal allele, but not shared with other pintails, though net divergence was only a single base substitution. These qualitative assessments seem more consistent with introgression rather than shared ancestral polymorphisms.

The STRUCTURE analysis yielded results consistent with low-level introgression and a single introgressed allele in each of the three aforementioned individuals, which had assignment probabilities of 0.959–0.961. These patterns were distinct from those appearing in a pintail x speckled teal F_1_ hybrid from the Falkland Islands that was identified using sequences from the same loci [17]. In the Falkland Islands study, the F_1_ hybrid and her offspring were identified with *P*  =  0.630 and *P*  =  0.860, respectively. The inferred level of introgression of speckled teal alleles into South Georgia pintails is thus low, identifiable by the presence of only a single heterospecific allele in each of three individuals. This suggests that the hybridization event(s) occurred several to many generations ago and that these three individuals have predominately South Georgia pintail genomes. However, unlike the case for hybridization within the Falkland Islands, the STRUCTURE analysis here does not unequivocally support hybridization as the cause of allele sharing.

The IMa2 model provides the ability to test these hypotheses using coalescent models of three or more populations. Because this analysis incorporates both divergence and gene flow in the same model, it is possible to quantitatively test whether shared alleles are due to introgression and gene flow or ancestral polymorphism. In this case, our analysis consisted of two populations of pintails (insular and continental), and a third population of speckled teal sampled throughout their continental distribution. Although we did not sample speckled teal on South Georgia due to their low abundance, the results indicate that gene flow has occurred into South Georgia pintails from speckled teal, as the relevant confidence interval did not overlap zero. In this case, the finding was aided by the serendipitous discovery of a single locus (ODC1) that had sorted to reciprocal monophyly and was divergent between the pintail and teal lineages. To the extent that more such loci can be identified, hybridization between these two insular populations could be studied at a wider genomic scale. Additionally, we observed no evidence that speckled teal mtDNA has introgressed into the South Georgia pintail population. Although this might be an artifact of small sample size, this finding is consistent with predictions from Haldane’s rule [15] and other recent findings that in hybrids the heterogametic sex (females in birds) is reproductively disadvantaged [60], [61], [62], [63], [64], [65].

Because we did not sample the speckled teal population on South Georgia and used the continental population in Argentina as a proxy, it is difficult to speculate about what levels of introgression have occurred into the speckled teal population, and whether the effects of hybridization have been bidirectional. It may be that introgression is still ongoing, or as our data suggest in the case of the pintails that it is not a recent event, but occurred some time ago. If this happened at a point in the distant past, it may have occurred at a time corresponding to different population sizes for both species, perhaps even during the colonization phase when both species were rare. Additional sampling including speckled teal from South Georgia would likely shed more light on the question of whether hybridization is ongoing or has ceased.

Finally, additional factors to consider are the locus-specific probability of detectability and influence of effective population size. Because ODC1 and PCK1 had sorted to reciprocal monophyly between the two species, it possible to identify heterospecific-origin alleles. For loci that have not sorted, however, and for which common alleles are still shared among species (e.g., ENO1, FGB, and GRIN1), these loci offer less utility. Evidence of interspecific gene flow also should be much easier to detect in the island population than in the continent because the continental populations have very large effective population sizes.

### Factors Leading to Hybridization

Interspecific hybridization is not uncommon in birds, especially the waterfowl. Indeed, the majority of known avian hybrids are represented by the Anatidae [13], and hybridization has been studied previously in a variety of waterfowl species using genetic assays [56], [66], [67], [68], [69], [70], [71]. Key factors contributing to hybridization in the waterfowl have been shown to include a tendency for hybridization to occur among more closely related species and between species that coexist in sympatry [12]. Randler [14], likewise, found that hybridization occurs more frequently when one species is common and the other species is rare, thus indicating that "scarcity of conspecifics facilitates hybridization in general." The findings that both pintails and speckled teal have hybridized on two separate island groups in the Subantarctic and South Atlantic Ocean, where their population ratios are imbalanced and oppositely skewed (as shown in this study and McCracken & Wilson [17]), thus provide further support for Hubbs' [16] "desperation hypothesis," which states that scarcity in one population and abundance of another will often lead to hybridization.

On South Georgia, pintails outnumber speckled teal approximately 300:1. The situation on this subantarctic island thus has the potential to facilitate hybridization because of uneven population sizes, a factor that likely contributed to hybridization between these two species in the Falkland Islands [17], where the ratios are reversed and speckled teal outnumber pintails 10:1 [72], [73], [74]. Finally, ARM collected two sibling pintail ducklings on South Georgia in 1997, one of which, as an adult female, possessed phenotypic characteristics (small size, dull bill, and metallic green in the speculum) similar to speckled teal. The sibling was normal. ARM has examined other pintails on South Georgia that possess varying degrees of metallic green in the speculum, whereas the majority of birds show no green at all. These observations suggest that additional evidence of interspecific hybridization (introgressed alleles) would likely be found on South Georgia.

### Development of Subantarctic Island Avifaunas

The finding that speckled teal alleles have introgressed into the South Georgia pintail population raises additional questions. For example, why is this particular speckled teal population breeding with pintails, and why has the speckled teal population not grown larger given their abundance and sympatry with pintails throughout continental habitats? Logic dictates that the pintails should have become established on South Georgia first, as they are more abundant in southern South America, better capable of dispersal, and their larger body size might make them better suited to colder environments. Because speckled teal are the next most abundant species in South America, logic also dictates that they would become the second waterfowl species to become established on South Georgia, first breeding on South Georgia possibly only a few decades before the first reports of their discovery in 1971 [38], [39]. Because South Georgia is still covered mostly in ice and is seasonally snow covered, both species must utilize the littoral environment. However, it may be that with a large pintail population already established in most of the suitable habitat on the island and new terrestrial habitat only slowly emerging from under the ice fields, niche space for speckled teal may be limited, though it should be noted that the density of ducks on South Georgia is not particularly high (ARM pers. obs.).

Availability of conspecific mates may present an additional problem for speckled teal at low population density. Hybridization is an important factor leading to population declines [3], [75], [76], and its effects could potentially be exacerbated in this island ecosystem because one waterfowl species is very common and the other is very rare. Small populations may thus suffer a type of inverse density dependence known as the Allee [18] effect, which can be described as a correlation between population density and the per-capita population growth rate in very small populations [77], [78], [79], [80]. Heterospecific pairing may thus be another factor restricting population growth as long as the speckled teal population persists below a critical population density. On the other hand, because the pintail population size is greater, the pintail population is expected to exhibit no Allee effect. Unless the speckled teal population density increases, the tendency to hybridize and backcross to the more common species may thus likely be high. In conclusion, the events unfolding between these duck species on South Georgia provide a unique window into two-species island population dynamics as they become established in a dynamic environment.
